# Diradicalar Character and Ring Stability of Mesoionic Heterocyclic Oxazoles and Thiazoles by Ab Initio Mono and Multi-Reference Methods

**DOI:** 10.3390/molecules25194524

**Published:** 2020-10-02

**Authors:** Antonio João da Silva Filho, Lucinêz da Cruz Dantas, Otávio Luís de Santana

**Affiliations:** Chemistry Department, Federal University of Paraiba, João Pessoa 58051-900, Brazil; antonio.sjf@gmail.com (A.J.d.S.F.); lucinezdantas@hotmail.com (L.d.C.D.)

**Keywords:** mesoionic, diradical, quantum-chemical calculations, single and multi-reference methods

## Abstract

Mesoionics are neutral compounds that cannot be represented by a fully covalent or purely ionic structure. Among the possible mesomeric structures of these compounds are the diradical electronic configurations. Theoretical and experimental studies indicate that some mesoionic rings are unstable, which may be related to a significant diradical character, that until then is not quantified. In this work, we investigated the diradical character of four heterocycles: 1,3-oxazol-5-one, 1,3-oxazol-5-thione, 1,3-thiazole-5-one, and 1,3-thiazole-5-thione. The oxazoles are known to be significatively less stable than thiazoles. DFT and ab initio single (B3LYP, MP2, CCSD, and QCISD) and ab initio multi-reference (MR-CISD) methods with three basis sets (6-311+G(d), aug-cc-pVDZ, and aug-cc-pVTZ) were employed to assess the diradical character of the investigated systems, in gas phase and DMSO solvent, from three criteria: (*i*) HOMO-LUMO energy gap, (*ii*) determination of energy difference between singlet and triplet wave functions, and (*iii*) quantification of the most significant diradical character (*y*_0_, determined in the unrestricted formalism). All of the results showed that the diradical character of the investigated systems is very small. However, the calculated electronic structures made it possible to identify the possible origin of the oxazoles instability, which can help the design of mesoionic systems with the desired properties.

## 1. Introduction

Heterocyclic compounds correspond to organic structures that have one or more different carbon atoms in the ring, with oxygen, nitrogen, and sulfur being the most common [1,2,3,4,5]. Normally the rings contain from three to six members, which makes it possible to obtain a huge variety of compounds, since a carbocyclic, regardless of its structure and functionality, can in principle be converted into analogous heterocyclic structures by replacing one or more endocyclic carbon atoms [6].

**Figure 1:**
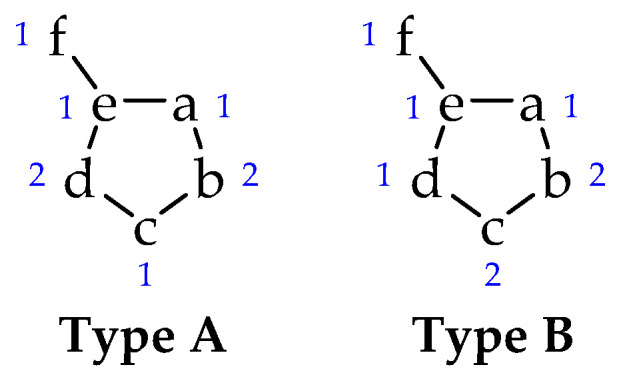
Type **A** and **B** of mesoionic structures. The numbers indicate the contribution of each atom to the *π* electron system.

Among a large number of heterocycles are mesoionic compounds [7,8], a class of molecules composed of five or six-membered rings, usually soluble in common organic solvents (such as chloroform and DMSO) [1,9] that can only be represented as mesomeric structures and that have a high charge separation. The mesoionics formed by five-membered rings are classified into two types (A and B), according to the contribution of each atom (or group) to the *π* electron system (Figure 1) [10,11]. Type A compounds are more stable (with a possible cleavage of the a-e bond) and have biological activity [12,13], while type B compounds undergo isomerization between their cyclic and acyclic forms (due to cleavage of the b-c bond) [14].

In mesoionic compounds the *p_z_* orbitals of the endocyclic atoms contribute a total of seven electrons and the *p_z_* orbital of the exocyclic atom contributes one. Thus, a sextet of *π* electrons can be obtained if one of the seven electrons of the ring pairing the single electron of the exocyclic atom, so that the mesoionic ring can, in principle, follow Hückel’s rule (4*n* + 2 electrons) and have an aromatic character, with a positive charge on the ring balanced by a negative charge located on the exocyclic atom. Although Simas et al. considered that mesoionic compounds are not aromatics [15,16,17], other investigations suggested that some types of mesoionic may have significant aromaticity [18,19,20]. Although the aromatic character contributes to stability, the lower aromaticity, or even the non-aromaticity, of heterocycles is not, by itself, the cause of the instability of some mesoionic systems, which may be related to a diradical character [21,22], derived from two electronic states with different multiplicities [23].

**Figure 2:**
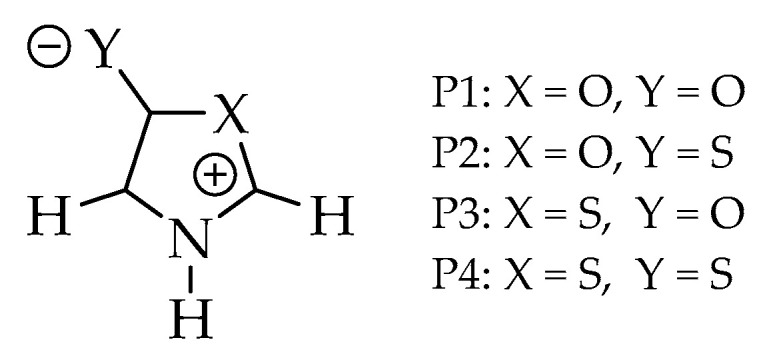
Structures of the investigated systems: Oxazoles (**P1** and **P2**) and thiazoles (**P3** and **P4**).

Five-membered nitrogenous heterocycles containing additional oxygen or sulfur heteroatoms, such as betaines, belong to a class of organic compounds called *azole* (Figure 2) [24]. These heterocycles are mesomeric structures that, in principle, discard the possibility of ring breakage. However, with advances in characterization techniques, it has been revealed that one of the mesoionic intra ring bond has a longer length, so that, by a thermal or photochemical effect, this bond, usually the C-O bond, may break, forming acyclic structures [25]. This instability of oxazoles was predicted by dos Anjos [17] and Champagne [26] through mono-reference calculations (DFT) and may be related to an until then unquantified diradical character. Similar theoretical calculations on 5-membered rings containing endocyclic sulfur are predicted to be stable systems [14,27].

In this work, we combined three criteria with DFT and ab initio single (B3LYP, MP2, CCSD, QCISD) and ab initio multi-reference (MR-CISD) methods with three basis sets (6-311 + G(d), aug-cc-pVDZ, and aug-cc-pVTZ) in gas phase and DMSO solvent to assess the diradical character of four mesoionic heterocycles: 1,3-oxazol-5-one (**P1**), 1,3-oxazol-5-thione (**P2**), 1,3-thiazole-5-one (**P3**), and 1,3-thiazole-5-thione (**P4**) (Figure 2). The goal of the computational calculation was to verify the possible relationship of the diradical character to the known instability of oxazole class. The investigated model systems, due to their small size, allow the use of MR-CISD method (reference method of the present work, with higher computational cost), making it possible, from a comparative study, to identify which mono-reference methods (of significantly lower computational cost) could be used with larger systems.

## 2. Models and Methods

### 2.1. Theoretical Model

The diradical character (*y*) is formally defined as twice the weight of the double excitation configuration in a multiconfigurational calculation (*y* = 2|*c*_D_|^2^, where *c*_D_ is the coefficient of the double excitation configuration from HOMO to LUMO) [28,29,30,31]. In cases where the diradical character is significant, the frontier molecular orbitals should be nearly degenerate, so there must be more than one determinant of significant weight in the description of the electronic configuration [32,33]. However, although multi-reference methods should preferably be employed for treating systems with strongly correlated electrons [34,35,36], diradical character has already been evaluated from spin-unrestricted mono-reference methods [37], such as DFT [38,39,40,41,42]. In the unrestricted formalism of mono-reference methods the diradical character can be evaluated from (*i*) the frontier orbital energy gaps [43,44,45], (*ii*) the energy difference between singlet and triplet wave functions [46,47], and (*iii*) the occupation numbers of frontier natural orbitals (HONO and LUNO), from which the multiple diradical characters *y_i_* are defined as [28,29,30,31,48,49,50,51,52,53]:

(1) $$y_{i}=1-\frac{2T_{i}}{1+T_{i}^{2}},\;T_{i}=\;\frac{1}{2}\left(n_{\mathrm{HONO}-i}-n_{\mathrm{LUNO}+i}\right),$$

where *T_i_* is the orbital overlap between the corresponding natural orbital pairs. According to Equation (1), the diradical character yi can range from 0 (closed-shell, when *n*_HONO−i_ = 2 and *n*_LUNO+i_ = 0) to 1 (pure open-shell, when *n*_HONO−i_ = 1 and *n*_LUNO+i_ = 1).

It is important to emphasize that there are other approximate strategies for investigating the diradical character, from the point of view of the formulation based on mono-reference methods [54], as well as the multi-reference electronic structure method of lower computational cost (such as the DFT/MRCI [55], which enables the consideration of other aspects, such as solvent effect and spin-orbit coupling), allowing the study of larger systems with more extensive basis sets. Although these strategies have not been used in the present work, they can be employed to test the hypothesis that the conclusions obtained with the model systems are transferable to larger systems, especially when in the presence of solvent.

### 2.2. Computational Methods

All single-reference calculations were performed with *Gaussian09* software [56], using standard frozen core configuration and standard convergence criteria for the B3LYP, MP2, QCISD, and CCSD electronic structure methods, with the 6-311 + G(d) and aug-cc-pVDZ basis set in gas phase and DMSO solvent. The solvent effect was taken into account using the C-PCM [57] continuum solvation model with standard cavitation and topological surface. The stationary points for the structures in the singlet electronic state were characterized as local minima by vibrational normal modes analysis. From the singlet state, all local minimum structures identified as cyclic were used to calculate the triplet state in the unrestricted formalism from single-point calculations with all methods (the triplet structures obtained from this procedure are identified as “rigid”, and the associated energy differences “vertical”). The structures were reoptimized in the triplet state, on the unrestricted formalism (UMP2, UQCISD, and UCCSD), to verify the geometry effect on the electronic properties (the triplet structures obtained from this procedure are identified as “relaxed”, and the associated energy differences “adiabatic”). This procedure makes it possible to evaluate the effect of structural changes, necessary for strategies with lower computational cost to treat larger systems. Two sets of additional calculations were performed to assess the effect of triple excitations and the basis set on the singlet-triplet energy differences in the gas-phase: CCSD(T) calculations were performed with 6-311 + G(d) and aug-cc-pVDZ (only for vertical singlet-triplet energy differences at CCSD geometries), and B3LYP and MP2 calculations were performed with aug-cc-pVTZ in the gas-phase (for vertical and adiabatic singlet-triplet energy differences).

Multi-reference calculations were performed with *Columbus* 7.0 program [58], using standard criteria for the MR-CISD method, with the 6-311+G(d) and aug-cc-pVDZ basis set in gas-phase. The heterocycles were characterized as minimum stationary points in the singlet wave function, and the triplet states were determined from single-point calculations. Singlet-triplet energy differences were calculated by including Davidson correction (MR-CISD+Q level) [59,60].

Theoretical and experimental studies of azoic systems (sydnones and munchnones) and analogs show that these structures have *C*_s_ symmetry [32,59,61,62,63]. For this reason, all calculations were performed imposing the symmetry constraint to minimize the computational cost. In the case of MR-CISD calculations, even small molecules under symmetry constraints can generate a configuration space with millions of CSFs, and some constraints on orbitals occupation in the CAS window need to be imposed. Thus, the inner layer orbitals were kept double occupied (frozen core - FC), the active molecular orbitals with occupations between zero and two were included in the active subspace (ACT, which includes ligands and *n*-type orbitals) and the corresponding anti-ligating orbitals (σ^*^_(C-X)_, π^*^_(C-C)_, and π^*^_(N-C)_) from the ACT subspace were placed in the auxiliary space (AUX) [58,59,60]. The orbitals used in this CASSCF(12,9) were: The non-bonding orbitals *n*_x(Y)_, and *n*_y(Y)_ (of the exocyclic atom), *n*_y(X)_ (of the endocyclic atom), the ligand orbitals *σ*_(C-X)_, *π*_(C-C)_, and *π*_(N-C)_ and the corresponding antibonding orbitals σ^*^_(C-X)_, π^*^_(C-C)_, and π^*^_(N-C)_ (see Supplementary Materials).

In this work, only the most prominent diradical character *y*_0_ was determined. In the case of mono-reference methods (B3LYP, MP2, QCISD, and CCSD) the *y*_0_ value was calculated according to Equation (1) from the occupation numbers of frontier natural orbitals (HONO and LUNO) obtained at the stationary point of the corresponding electronic structure method [28,29,30,31,48,49,50,51,52,53]. For the multi-reference method (MR-CISD) the *y*_0_ was determined from the weight of the double excitation configuration (from HOMO to LUMO) [28,29,30,31].

## 3. Results and Discussion

One of the parameters used to analyze the diradical character of heterocyclics from mono-reference methods (the HOMO-LUMO gap, *E*_HL_) is presented in Table 1 (as shown in Supplementary Materials, the HOMO is of *a*″ symmetry and the LUMO of *a*′ symmetry). As pointed out by Baerends et al., the gap of Kohn-Sham frontier orbitals has a different meaning from the gap associated with the Hartree-Fock frontier orbitals, which is why they are not included in Table 1 [64]. The energy gap was calculated from Hartree-Fock molecular orbitals obtained at the stationary point of the corresponding electronic structure method (due to this, only small differences are expected between different methods). The smaller the difference, the greater the diradical character.

According to the results of Table 1, the smallest HOMO-LUMO gap occurs for the 1,3-oxazol-5-thione (**P2**) and 1,3-thiazole-5-thione (**P4**) in the gas phase (approximately 7.9 and 7.4 eV with 6-311+G(d) and aug-cc-pVDZ basis set, respectively). These values are significantly high, which indicates (qualitatively) a very small diradical character [65,66]. In all cases, the solvent effect contributes to increase the HOMO-LUMO gap, this effect being more significant than the associated with the increase of the basis set (see Supplementary Materials).

Table 2 shows the values for the singlet-triplet energy difference (*E*_ST_) obtained from the rigid (triplet values in the singlet geometries) and relaxed (optimized geometries in the triplet state, except with the MR-CISD method) geometries. In all cases, the results have a negligible basis set dependence, with the triplet electronic state having higher energy (positive values for vertical and adiabatic *E*_ST_).

As shown in Table 2, a significant decrease in *E*_ST_ values is observed with geometry relaxation in all single-reference methods (in gas phase and DMSO solvent). The MP2 (vertical and adiabatic) and MR-CISD+Q (vertical) results are systematically higher than those obtained with the other methods for all structures. The smallest adiabatic singlet-triplet energy differences for investigated structures were obtained for the 1,3-oxazol-5-thione (**P2**) and 1,3-thiazole-5-thione (**P4**) heterocycles, but with very high values (*E*_ST_ > 1.3 eV) to indicate a diradical character (well above the *k*_B_*T* value) [63,67]. In all cases, the solvent effect contributes to increase the singlet-triplet energy differences, this effect being more significant than associated with the increase of the basis set (in particular, the adiabatic *E*_ST_ is increased in all cases; see Supplementary Materials). It is important to note that the effect of triple excitations is to reduce the vertical singlet-triplet energy differences (in the gas phase), although the final energies are still very high (*E*_ST_ > 1.5 eV).

The diradical character of heterocycles, quantified by the *y*_0_ parameter, determined from Equation (1) in the case of single-reference methods, and from the weight of the double excitation configuration for MR-CISD calculation, is summarized in Table 3 (as shown in Supplementary Materials for multi-reference calculations, the ground state is of 1^1^ A′ symmetry for all structures and the first excited state is of 2^1^ A′ for **P1** and **P3** systems, and a mixture of 2^1^ A′ and 1^1^ A″ for **P2** and **P4** structures). Again, the results are almost independent of the basis set (including the aug-cc-pVTZ basis set; see Supplementary Materials). According to this parameter, the diradical character, from the single-reference methods (B3LYP, MP2, QCISD, and CCSD), in any case, is less than 1.0% (in gas phase and DMSO solvent), which indicates (quantitatively) a very small diradical character. This result is consistent with that obtained by the MR-CISD method, for which the diradical character does not reach 2.5%. It is important to note that, although the numerical differences between the *y*_0_ values can be attributed to the differences in accuracy between the mono and multi-reference methods employed, in all cases a negligible diradical character is predicted, and any ordering of the diradical character between different structures become meaningless.

All the criteria employed so far have shown that the investigated heterocycles have a negligible diradical character, and therefore, the instability of oxazoles should be attributed to another structural property. Aiming to identify the source of instability from the analysis of other electronic properties of these systems, the dipole moments of heterocyclic compounds were determined (in gas phase and DMSO solvent) by the five electronic structure methods (Table 4), with the aug-cc-pVDZ basis set. Dipole moments were determined from restricted singlet (*μ*_1r_) and unrestricted triplet (*μ*_3u_) wave functions. For the triplet case, the rigid (to identify the effect of the wave function used) and relaxed (optimized, except with MR-CISD method) geometries were considered. In all cases, results are almost independent of the basis set (see Supplementary Materials for results with 6-311 + G(d) and aug-cc-pVTZ basis sets).

Considering the effect of the wave function, it is observed that the singlet electronic state (in gas phase and DMSO solvent) shows the highest dipole moments, which indicates a greater charge separation, consistent with the elevated mesoionic character [7,8]. In all cases, the solvent contributes to increase the dipole moment in all electronic states. The lowest dipole moment was identified for the 1,3-thiazole-5-one (**P3**) structure (on average 6.5 D in gas phase and 10.4 D in DMSO solvent for *μ*_1r_, within the characteristic range for mesoionic compounds, 5–15 D) [68]. Moreover, the effect of geometry relaxation in the triplet state is to systematically reduce the dipole moment at different levels of theory. In general, the single-reference QCISD and CCSD results (in gas phase), regardless of basis set, were closer to MR-CISD values.

**Figure 3:**
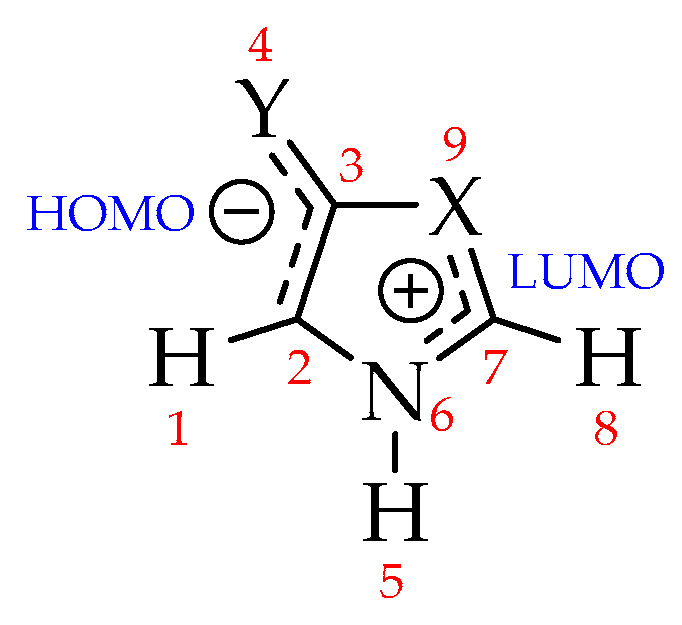
Expected charge separation for mesoionic compounds [15].

Mesoionics are neutral structures with high charge separation, and whose formal charges are located in two well-defined regions: One, negatively charged (where HOMO is located), is associated with exocyclic atoms, and another, positively charged (where LUMO is located), associated with endocyclic atoms (Figure 3) [63,69,70,71]. The charges on the exo (atoms 1–4) and endocyclic (atoms 5–9) atomic groups were investigated and the results are summarized in Table 5, referring to Natural Bond Orbitals (NBO) charge partitioning [72,73,74] (see Supplementary Materials for the full set of results).

**Figure 4:**
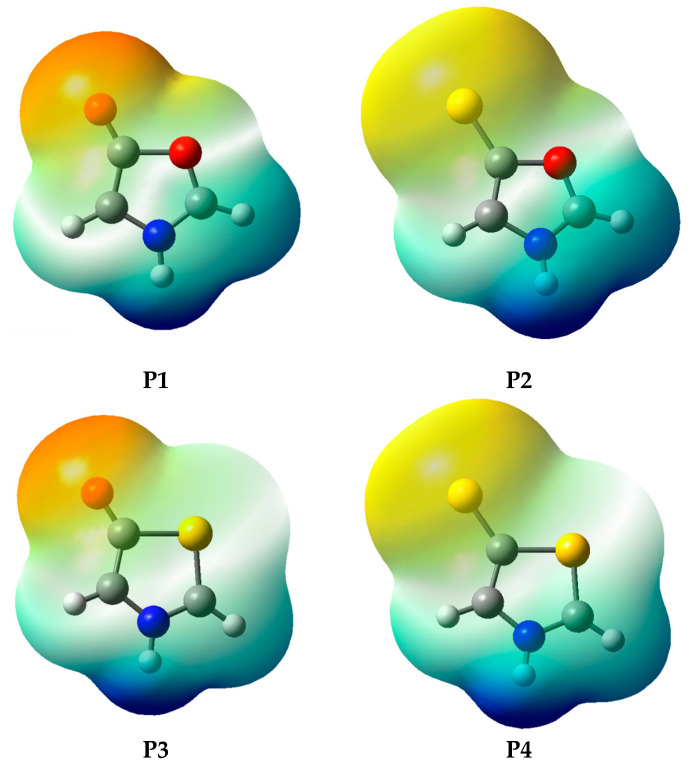
Electrostatic potential map, calculated at the MP2/aug-cc-pVDZ level.

According to the results in Table 5, although all investigated systems have the expected behavior with respect to charge separation [7,8], it is observed that the **P1** and **P2** systems have lower negative charges in the exocyclic atomic group. In these systems, part of the negative charge is shifted to the endocyclic oxygen atom (position 9, Figure 3), which is attributed to its high electronegativity. This charge shift can be verified from the electrostatic potential map (Figure 4), obtained at the MP2/aug-cc-pVDZ level (this result has a negligible dependence on the electronic structure method; see Supplementary Materials for the full set of results). Thus, the high electronegativity of oxygen in oxazoles results in structures that, although fitting the mesoionic model for global charge separation, may have their known instability [17,25,26] related to a purely electrostatic effect, so that the partial charges with the same sign at atoms Y_4_ and O_9_ may weaken the bond between atoms C_3_ and O_9_. In systems **P3** and **P4** the partial charges on atoms Y_4_ and S_9_ have different signs, leading to the opposite effect. It is important to note that the solvent contributes to increase the charge separation, without changing the signals of the charges on endo and exocyclic atoms observed in the gas phase.

## 4. Conclusions

In this work, we investigate the nature of the lower stability of oxazoles compared to thiazoles. The hypothesis was that the lower stability of oxazoles is due to a significant diradical character, that until then is not quantified. The diradical character was evaluated using mono (B3LYP, MP2, CCSD, and QCISD, in gas phase and DMSO solvent) and multi-reference (MR-CISD, in gas phase) methods, considering three criteria: (*i*) The frontier orbital energy gaps, (*ii*) the energy difference between singlet and triplet wave functions, and (*iii*) the diradical character *y*_0_.

The reduction in the HOMO and LUMO energy gap implies an increase in the diradical character. Although this criterion is not sufficient to quantify the diradical character, it is interesting to note that the smallest observed gaps in this work (heterocyclic 1,3-thiazole-5-thione, with 7.4 eV < *E*_HL_ < 7.8 eV in gas phase) correspond to very high values, suggesting that these structures do not have a significant diradical character. This same conclusion is derived, with all mono and multi-reference methods, from the analysis of the singlet-triplet energy difference (whose smallest adiabatic values are greater than 1.3 eV in the gas phase, well above the typical values for *k*_BT_) and the *y*_0_ parameter (whose values are less than 2.5% for all systems), indicating that investigated heterocyclics have a small diradical character. These results, combined with similar conclusions from the literature, suggest that methods with lower computational cost (in particular, mono and multi-reference DFT based methods) can be used in the study of larger mesoionic systems, with substituent groups, as well as the consideration of additional aspects, such as the solvent effect.

The values obtained for dipole moments are consistent with those expected for mesoionic structures (*μ* > 5 D). However, although charge separation is consistent with that expected for a mesoionic compound, in oxazoles (**P1** and **P2**) the endo X and exocyclic Y atoms have partial charges of the same sign, which may be related to the instability of this class of heterocyclics.

## Figures and Tables

**Table 1:** HOMO-LUMO (*E*_HL_) energy gap (in eV) for the investigated systems in gas phase and DMSO solvent.

Method	P1		P2		P3		P4	
	BS1 *^a^*	BS2 *^b^*	BS1 *^a^*	BS2 *^b^*	BS1 *^a^*	BS2 *^b^*	BS1 *^a^*	BS2 *^b^*
**Gas Phase**								
**MP2**	8.89	8.21	7.94	7.35	8.68	8.15	7.82	7.35
**QCISD**	8.98	8.31	7.95	7.37	8.78	8.26	7.82	7.37
**CCSD**	8.95	8.28	7.94	7.36	8.77	8.24	7.81	7.37
**DMSO Solvent**								
**MP2**	9.96	9.09	9.60	8.89	9.63	8.96	9.36	8.82
**QCISD**	10.06	9.20	9.69	8.99	9.78	9.13	9.50	8.93
**CCSD**	10.05	9.18	9.69	8.99	9.77	9.11	9.51	8.93

*^a^* BS1: Basis Set 6-311 + G(d). *^b^* BS2: Basis Set aug-cc-pVDZ.

**Table 2:** Singlet-triplet (*E*_ST_) energy differences (in eV) for the investigated systems in the gas phase and DMSO solvent.

Method	P1		P2		P3		P4	
	BS1 *^a^*	BS2 *^b^*	BS1 *^a^*	BS2 *^b^*	BS1 *^a^*	BS2 *^b^*	BS1 *^a^*	BS2 *^b^*
**Vertical/Gas Phase**								
**B3LYP**	---*^d^*	2.89	2.37	2.33	2.40	2.36	1.90	1.89
**MP2**	3.31	3.31	2.93	2.99	3.05	3.07	2.57	2.66
**QCISD**	2.83	2.80	2.35	2.36	2.30	2.27	1.81	1.83
**CCSD**	2.77	2.75	2.33	2.36	2.28	2.26	1.81	1.84
**MRCI** *^c^*	3.29	3.34	2.86	2.86	2.73	2.74	2.19	2.24
**Vertical/DMSO Solvent**								
**B3LYP**	---*^d^*	---*^d^*	2.83	2.80	2.55	2.51	2.33	2.30
**MP2**	3.46	3.44	3.40	3.46	3.17	3.16	2.93	3.00
**QCISD**	3.04	3.00	2.95	2.97	2.56	2.51	2.38	2.39
**CCSD**	3.03	2.99	2.96	2.50	2.55	2.42	2.40	2.99
**Adiabatic/Gas Phase**								
**B3LYP**	---*^d^*	1.89	1.61	1.64	1.87	1.88	1.53	1.55
**MP2**	2.30	2.38	2.15	2.30	2.53	2.61	2.12	2.26
**QCISD**	1.68	1.72	1.46	1.53	1.70	1.72	1.37	1.42
**CCSD**	1.66	1.70	1.45	1.53	1.68	1.71	1.35	1.42
**Adiabatic/DMSO Solvent**								
**B3LYP**	---*^d^*	---*^d^*	2.10	2.33	2.07	2.12	1.94	1.99
**MP2**	2.52	2.89	2.60	2.94	2.65	2.82	2.46	2.63
**QCISD**	2.01	2.34	2.08	2.43	1.96	2.08	1.90	2.03
**CCSD**	2.00	2.33	2.08	2.44	1.95	2.07	1.91	2.04

*^a^* BS1: Basis Set 6-311 + G(d). *^b^* BS2: Basis Set aug-cc-pVDZ. *^c^* MRCI: MR-CISD+Q. *^d^* The structure opens during optimization.

**Table 3:** Percentage values of *y*_0_ for the investigated heterocycles in gas phase and DMSO solvent.

Method	P1		P2		P3		P4	
	Rigid	Relax.	Rigid	Relax.	Rigid	Relax.	Rigid	Relax.
**6-311 + G(d)/Gas Phase**								
**B3LYP**	0.00	0.00	0.00	0.00	0.00	0.00	0.00	0.00
**MP2**	0.23	0.23	0.24	0.24	0.09	0.35	0.10	0.33
**QCISD**	0.40	0.40	0.42	0.42	0.20	0.21	0.22	0.54
**CCSD**	0.32	0.32	0.33	0.33	0.18	0.18	0.20	0.39
**MRCI** *^a^*	---*^b^*	2.36	---*^b^*	1.61	---*^b^*	1.87	---*^b^*	2.03
**aug-cc-pVDZ/Gas Phase**								
**B3LYP**	0.00	0.00	0.00	0.00	0.00	0.00	0.00	0.00
**MP2**	0.25	0.25	0.26	0.26	0.11	0.37	0.12	0.35
**QCISD**	0.42	0.42	0.44	0.44	0.22	0.23	0.24	0.56
**CCSD**	0.34	0.34	0.35	0.35	0.20	0.20	0.22	0.41
**MRCI** *^a^*	---*^b^*	1.53	---*^b^*	1.64	---*^b^*	1.67	---*^b^*	1.95
**6-311 + G(d)/DMSO Solvent**								
**B3LYP**	---*^c^*	---*^c^*	0.00	0.00	0.00	0.00	0.00	0.00
**MP2**	0.21	0.21	0.21	0.21	0.12	0.27	0.25	0.25
**QCISD**	0.35	0.35	0.29	0.34	0.26	0.42	0.38	0.38
**CCSD**	0.29	0.29	0.29	0.29	0.20	0.34	0.33	0.33
**aug-cc-pVDZ/DMSO Solvent**								
**B3LYP**	---*^c^*	---*^c^*	0.00	0.00	0.00	0.00	0.00	0.00
**MP2**	0.23	0.23	0.24	0.24	0.13	0.29	0.18	0.27
**QCISD**	0.37	0.37	0.36	0.36	0.25	0.45	0.23	0.41
**CCSD**	0.31	0.31	0.31	0.31	0.21	0.36	0.21	0.35

*^a^* MRCI: MR-CISD. *^b^* Only singlet optimized geometries. *^c^* The structure opens during optimization.

**Table 4:** Dipole moments (*μ*), in Debye (D), for singlet (*μ*_1r_) and triplet (*μ*_3u_) wave functions (rigid/relaxed), with aug-cc-pVDZ basis set in gas phase and DMSO solvent.

Method	P1		P2		P3		P4	
	*μ*_1r_	*μ*_3u_	*μ*_1r_	*μ*_3u_	*μ*_1r_	*μ*_3u_	*μ*_1r_	*μ*_3u_
**Gas Phase**								
**B3LYP**	6.91	5.22/4.78	8.66	4.82/4.37	6.34	5.16/4.54	8.02	4.91/4.45
**MP2**	6.99	6.03/5.17	8.69	5.47/4.99	6.24	6.13/5.13	7.93	5.78/5.23
**QCISD**	7.27	5.22/4.86	9.29	4.14/3.93	6.68	5.13/4.40	8.71	4.34/3.97
**CCSD**	7.39	5.22/4.85	9.41	4.14/3.86	6.73	5.14/4.52	8.83	4.35/3.96
**MRCI** *^a^*	7.28	5.48 *^b^*	9.23	4.82 *^b^*	6.57	5.41 *^b^*	8.31	4.60 *^b^*
**DMSO Solvent**								
**B3LYP**	---*^c^*	---*^c^*/7.25	13.07	6.95/7.50	9.14	7.25/7.10	12.77	7.09/7.39
**MP2**	11.15	7.16/7.29	14.72	5.85/7.51	10.66	7.06/7.03	14.70	6.17/7.48
**QCISD**	11.23	7.04/7.26	14.91	5.56/6.84	10.89	6.91/6.94	15.00	5.85/6.74
**CCSD**	11.23	7.04/7.24	14.92	5.56/6.84	10.86	5.56/6.94	15.00	5.85/6.75

*^a^* MRCI: MR-CISD. *^b^* Only triplet rigid geometries. *^c^* The structure opens during optimization.

**Table 5:** NBO charges on exo and endocyclic atom groups on the aug-cc-pVDZ basis set. Values in parentheses correspond to the partial charges of exo Y and endo X atoms.

Structure *^a^*	Method	Endo Atoms	Exo Atoms
**Gas Phase**			
**P1**	**B3LYP**	+0.008 (−0.541)	−0.008 (−0.604)
	**MP2**	+0.004 (−0.640)	−0.005 (−0.757)
	**QCISD**	+0.017 (−0.642)	−0.017 (−0.756)
	**CCSD**	+0.019 (−0.637)	−0.019 (−0.759)
**P2**	**B3LYP**	+0.061 (−0.480)	−0.061 (−0.188)
	**MP2**	+0.077 (−0.573)	−0.077 (−0.300)
	**QCISD**	+0.099 (−0.573)	−0.100 (−0.321)
	**CCSD**	+0.101 (−0.570)	−0.100 (−0.323)
**P3**	**B3LYP**	+0.245 (+0.248)	−0.245 (−0.607)
	**MP2**	+0.318 (+0.275)	−0.317 (−0.761)
	**QCISD**	+0.336 (+0.256)	−0.335 (−0.764)
	**CCSD**	+0.335 (+0.259)	−0.334 (−0.762)
**P4**	**B3LYP**	+0.437 (+0.441)	−0.437 (−0.179)
	**MP2**	+0.509 (+0.467)	−0.509 (−0.302)
	**QCISD**	+0.536 (+0.458)	−0.536 (−0.329)
	**CCSD**	+0.538 (+0.463)	−0.537 (−0.329)
**DMSO Solvent**			
**P1**	**B3LYP**	---*^b^*	---*^b^*
	**MP2**	+0.139 (−0.636)	−0.139 (−0.871)
	**QCISD**	+0.125 (−0.636)	−0.125 (−0.874)
	**CCSD**	+0.126 (−0.634)	−0.126 (−0.875)
**P2**	**B3LYP**	+0.200 (−0.479)	−0.200 (−0.416)
	**MP2**	+0.215 (−0.575)	−0.215 (−0.513)
	**QCISD**	+0.226 (−0.576)	−0.226 (−0.527)
	**CCSD**	+0.227 (−0.575)	−0.227 (−0.529)
**P3**	**B3LYP**	+0.380 (+0.280)	−0.380 (−0.718)
	**MP2**	+0.475 (+0.312)	−0.475 (−0.872)
	**QCISD**	+0.490 (+0.289)	−0.490 (−0.877)
	**CCSD**	+0.492 (+0.294)	−0.492 (−0.876)
**P4**	**B3LYP**	+0.630 (+0.490)	−0.630 (−0.412)
	**MP2**	+0.706 (+0.507)	−0.706 (−0.526)
	**QCISD**	+0.714 (+0.486)	−0.714 (−0.541)
	**CCSD**	+0.715 (+0.489)	−0.715 (−0.542)

*^a^* Optimized geometries. *^b^* The structure opens during optimization.

## Supplementary Materials

The following are available online.

## References

[1] Joule J.A., Mills K. (2010). Heterocyclic Chemistry.

[2] Joule J.A., Mills K. (2013). Heterocyclic Chemistry at a Glance.

[3] Fraser W. (2009). Comprehensive Heterocyclic Chemistry II: A Review of the Literature 1982–1995.

[4] Martins P., Jesus J., Santos S., Raposo L., Roma-Rodrigues C., Baptista P. (2015). Heterocyclic Anticancer Compounds: Recent Advances and the Paradigm Shift Towards the Use of Nanomedicine’s Tool Box. Molecules.

[5] Kalaria P.N., Karad S.C., Raval D.K. (2018). A review on diverse heterocyclic compounds as the privileged scaffolds in antimalarial drug discovery. Eur. J. Med. Chem..

[6] Hossain M., Nanda A.K. (2018). A Review on Heterocyclic: Synthesis and Their Application in Medicinal Chemistry of Imidazole Moiety. Sci. J. Chem..

[7] Bhosale S.K., Deshpande S.R., Wagh R.D. (2012). Mesoionic Sydnone Derivatives: An Overview. J. Chem. Pharm. Res..

[8] Nič M., Jirát J., Košata B., Jenkins A., McNaught A. (2014). IUPAC Gold Book: Compendium of Chemical Terminology.

[9] Asif M. (2016). A Review on Phamacological Potentials of Various Substituted Thiadiazole Analogs. Int. J. Curr. Res. Appl. Chem. Chem. Eng..

[10] Schmidt A., Wiechmann S., Freese T. (2013). Recent advances in neutral and anionic N-heterocyclic carbene—Betaine interconversions. synthesis, characterization, and applications. Arkivoc.

[11] Moderhack D. (2016). Mesoionic Tetrazoles Progress Since 1980. Heterocycles.

[12] Ollis W.D., Ramsden C.A. (1976). Meso-ionic Compounds. Adv. Heterocycl. Chem..

[13] Abdualkader A.M., Taher M., Yusoff N.I.N. (2017). Mesoionic Sydnone: A Review in Their Chemical and Biological Properties. Int. J. Pharm. Pharm. Sci..

[14] Ramsden C.A., Oziminski W.P. (2019). An ab initio study of the valence tautomerism of type B mesoionic rings. Tetrahedron Lett..

[15] de Oliveira M.B., Miller J., Pereira A.B., Galembeck S.E., de Moura G.L.C., Simas A.M. (1996). Mesoionic 2-N-cycloalkylamino-5-alkyl-1,3-dithiolium-4-thiolates. Phosphorus Sulfur Silicon Relat. Elem..

[16] Simas A.M., Miller J., de Athayde Filho P.F. (1998). Are mesoionic compounds aromatic?. Can. J. Chem..

[17] Anjos I.C., Vasconcellos M.L.A.A., Rocha G.B. (2012). A DFT and Natural Resonance Theory investigation of the electronic structure of mesoionic compounds. Theor. Chem. Acc..

[18] Badami B.V. (2006). Mesoionic Compounds. Resonance.

[19] Nein Y.I., Morzherin Y.Y. (2012). Criteria for aromaticity of mesoionic heterocycles. Russ. Chem. Bull..

[20] Oziminski W.P., Ramsden C.A. (2015). A DFT and ab initio study of conjugated and semi-conjugated mesoionic rings and their covalent isomers. Tetrahedron.

[21] Srinivas K., Prabhakar C., Devi C.L., Yesudas K., Bhanuprakash K., Rao V.J. (2007). Enhanced diradical nature in oxyallyl derivatives leads to near infra red absorption: A comparative study of the squaraine and croconate dyes using computational techniques. J. Phys. Chem. A.

[22] Prabhakar C., Yesudas K., Bhanuprakash K., Rao V.J., Santosh Kumar R.S., Rao D.N. (2008). Linear and Nonlinear Optical Properties of Mesoionic Oxyallyl Derivatives: Enhanced Non-Resonant Third Order Optical Nonlinearity in Croconate Dyes. J. Phys. Chem. C.

[23] Abe M. (2013). Diradicals. Chem. Rev..

[24] Kakkar S., Narasimhan B. (2019). A comprehensive review on biological activities of oxazole derivatives. BMC Chem..

[25] Veedu R.N., Kvaskoff D., Wentrup C. (2014). Sydnone Photochemistry: Direct Observation of Earl’s Bicyclic Lactone Valence Isomers (Oxadiazabicyclo[2.1.0] pentanones), Formation of Carbodiimides, Reaction Mechanism, and Photochromism. Aust. J. Chem..

[26] Champagne P.A., Houk K.N. (2017). Influence of Endo- and Exocyclic Heteroatoms on Stabilities and 1,3-Dipolar Cycloaddition Reactivities of Mesoionic Azomethine Ylides and Imines. J. Org. Chem..

[27] Fabian J., Hess B.A. (2002). Sulfur-containing mesoionic compounds: Theoretical study on structure and properties. Int. J. Quant. Chem..

[28] Yamanaka S., Okumura M., Nakano M., Yamaguchi K. (1994). EHF theory of chemical reactions Part 4. UNO CASSCF, UNO CASPT2 and R(U)HF coupled-cluster (CC) wavefunctions. J. Mol. Struct..

[29] Nakano M., Champagne B. (2015). Theoretical Design of Open-Shell Singlet Molecular Systems for Nonlinear Optics. J. Phys. Chem. Lett..

[30] Nakano M. (2014). Excitation Energies and Properties of Open-Shell Singlet Molecules.

[31] Sarto M.S., De Bellis G., Tamburrano A., D’Aloia A.G., Marra F. (2016). Graphene Science Handbook: Electrical and Optical Properties.

[32] Kishi R., Bonness S., Yoneda K., Takahashi H., Nakano M., Botek E., Champagne B., Kubo T., Kamada K., Ohta K. (2010). Long-range corrected density functional theory study on static second hyperpolarizabilities of singlet diradical systems. J. Chem. Phys..

[33] Nakano M., Kishi R., Ohta S., Takebe A., Takahashi H., Furukawa S.I., Kubo T., Morita Y., Nakasuji K., Yamaguchi K. (2006). Origin of the enhancement of the second hyperpolarizability of singlet diradical systems with intermediate diradical character. J. Chem. Phys..

[34] Crawford T.D., Kraka E., Stanton J.F., Cremer D. (2001). Problematic p-benzyne: Orbital instabilities, biradical character, and broken symmetry. J. Chem. Phys..

[35] Barone V., Cacelli I., Ferretti A., Montic S., Prampolini G. (2011). Singlet-triplet energy gap of a diarylnitroxide diradical by an accurate many-body perturbative approach. Phys. Chem. Chem. Phys..

[36] Das A., Müller T., Plasser F., Lischka H. (2016). Polyradical Character of Triangular Non-Kekulé Structures, Zethrenes, p-Quinodimethane-Linked Bisphenalenyl, and the Clar Goblet in Comparison: An Extended Multireference Study. J. Phys. Chem. A.

[37] Houk K.N., Beno B.R., Nendel M., Black K., Yoo H.Y., Wilsey S., Lee J.K. (1997). Exploration of pericyclic reaction transition structures by quantum mechanical methods: Competing concerted and stepwise mechanisms. J. Mol. Struct..

[38] Ovchinnikov A.A., Labanowski J.K. (1996). Simple spin correction of unrestricted density-functional calculation. Phys. Rev. A.

[39] Markovič S., Durdević J., Jeremić S., Gutman I. (2010). Diradical character of some fluoranthenes. J. Serb. Chem. Soc..

[40] Fukui H., Shigeta Y., Nakano M., Kubo T., Kamada K., Ohta K., Champagne B., Botek E. (2011). Enhancement of second hyperpolarizabilities in open-shell singlet slipped-stack dimers composed of square planar nickel complexes involving o-semiquinonato type ligands. J. Phys. Chem. A.

[41] Xu X., Gozem S., Olivucci M., Truhlar D.G. (2013). Combined Self-Consistent-Field and Spin-Flip Tamm-Dancoff Density Functional Approach to Potential Energy Surfaces for Photochemistry. J. Phys. Chem. Lett..

[42] Canola S., Casado J., Negri F. (2018). The double exciton state of conjugated chromophores with strong diradical character: Insights from TDDFT calculations. Phys. Chem. Chem. Phys..

[43] Salem L., Rowland C. (1972). The Electronic Properties of Diradicals. Angew. Chem. Int. Ed..

[44] Michl J., Bonačić-Koutecký V. (1988). Biradicals and Biradicaloids: A Unified View. Tetrahedron.

[45] Ichimura A.S., Lahti P.M., Matlin A.R. (1990). Ab Initio Computational Study of Methano- and Ethano-Bridged Derivatives of Oxyallyl. J. Am. Chem. Soc..

[46] Krylov A.I. (2017). The Quantum Chemistry of Open-Shell Species. Rev. Comp. Chem..

[47] Barone V., Cacelli I., Ferretti A. (2018). The role of the multiconfigurational character of nitronyl-nitroxide in the singlet-triplet energy gap of its diradicals. Phys. Chem. Chem. Phys..

[48] Qiu Y.Q., Wang W.Y., Ma N.N., Wang C.H., Zhang M.Y., Zou H.Y., Liu P.J. (2013). Computational investigation on redox-switchable nonlinear optical properties of a series of polycyclic p-quinodimethane molecules. J. Mol. Model..

[49] Rivero P., Jiménez-Hoyos C.A., Scuseria G.E. (2013). Entanglement and polyradical character of polycyclic aromatic hydrocarbons predicted by projected Hartree-Fock theory. J. Phys. Chem. B.

[50] Ramos-Cordoba E., Salvador P. (2014). Diradical character from the local spin analysis. Phys. Chem. Chem. Phys..

[51] Mondal A., Hatua K., Nandi P.K. (2015). Static second hyperpolarizability of twisted ethylene: A comprehensive computational study. J. Theor. Comput. Chem..

[52] Gopalakrishna T.Y., Zeng W., Lu X., Wu J. (2018). From open-shell singlet diradicaloids to polyradicaloids. Chem. Commun..

[53] Tobe Y. (2018). Quinodimethanes Incorporated in Non-Benzenoid Aromatic or Antiaromatic Frameworks. Top. Curr. Chem..

[54] Sugisaki K., Nakazawa S., Toyota K., Sato K., Shiomi D., Takui T. (2019). Quantum Chemistry on Quantum Computers: A Method for Preparation of Multiconfigurational Wave Functions on Quantum Computers without Performing Post-Hartree-Fock Calculations. ACS Cent. Sci..

[55] Tatchen J., Kleinschmidt M., Marian C.M. (2004). Electronic excitation spectra and singlet-triplet coupling in psoralen and its sulfur and selenium analogs. J. Photochem. Photobiol. A Chem..

[56] Frisch M.J., Trucks G.W., Schlegel H.B., Scuseria G.E., Robb M.A., Cheeseman J.R., Scalmani G., Barone V., Mennucci B., Petersson G.A. (2009). Gaussian 09 A.02.

[57] Cossi M., Rega N., Scalmani G., Barone V. (2003). Energies, structures, and electronic properties of molecules in solution with the C-PCM solvation model. J. Comp. Chem..

[58] Lischka H., Shepard R., Shavitt I., Pitzer R.M., Dallos M., Müller T., Szalay P.G., Brown F.B., Ahlrichs R., Böhm H.J. (2017). COLUMBUS: An. Ab-Initio Electronic Structure Program. Release 7.0.

[59] Antol I., Eckert-Maksic M., Lischka H. (2004). Ab initio MR-CISD study of gas-phase basicity of formamide in the first excited singlet state. J. Phys. Chem. A.

[60] Lischka H., Müller T., Szalay P.G., Shavitt I., Pitzer R.M., Shepard R. (2011). COLUMBUS—A program system for advanced multireference theory calculations. WIREs Comput. Mol. Sci..

[61] de Morais S.A., da Silva Morais C.R., de Athayde Filho P.F., Freitas Lira B., do Nascimento R.S.T.R. (2009). A kinetic study of the thermal decomposition of mesoionic compounds within scope of its application in nonlinear optical devices. J. Therm. Anal. Calorim..

[62] Nunes C.M., Reva I., Pinho E., Melo T.M.V.D., Fausto R., Šolomek T., Bally T. (2011). The pyrolysis of isoxazole revisited: A new primary product and the pivotal role of the vinylnitrene. A low-temperature matrix isolation and computational study. J. Am. Chem. Soc..

[63] Beker W., Szarek P., Komorowski L., Lipiński J. (2013). Reactivity patterns of imidazole, oxazole, and thiazole as reflected by the polarization justified Fukui functions. J. Phys. Chem. A.

[64] Baerends E.J., Gritsenko O.V., Van Meer R. (2013). The Kohn-Sham gap, the fundamental gap and the optical gap: The physical meaning of occupied and virtual Kohn-Sham orbital energies. Phys. Chem. Chem. Phys..

[65] Prabhakar C., Chaitanya G.K., Sitha S., Bhanuprakash K., Rao V.J. (2005). Role of the oxyallyl substructure in the Near Infrared (NIR) absorption in symmetrical dye derivatives: A computational study. J. Phys. Chem. A.

[66] Yesudas K., Bhanuprakash K. (2007). Origin of near-infrared absorption and large second hyperpolarizability in oxyallyl diradicaloids: A three-state model approach. J. Phys. Chem. A.

[67] Wirz J. (1984). Spectroscopic and kinetic investigations of conjugated biradical intermediates. Pure Appl. Chem..

[68] Mathieu S., Trinquier G. (2019). Oxidative addition of carbon dioxide into mesoionics. Phys. Chem. Chem. Phys..

[69] Fonseca T.L., de Oliveira H.C.B., Castro M.A. (2008). Theoretical study of the lowest electronic transitions of sulfur-bearing mesoionic compounds in gas-phase and in dimethyl sulfoxide. Chem. Phys. Lett..

[70] Bosco C.A.C., Maciel G.S., Rakov N., de Araújo C.B., Acioli L.H., Simas A.M., Athayde-Filho P.F., Miller J. (2007). Probing the nuclear susceptibility of mesoionic compounds using two-beam coupling with chirp-controlled pulses. Chem. Phys. Lett..

[71] Moura G.L.C., Simas A.M. (2008). Two-photon absorption cross-sections from electronic structure methods: Mesoionic compounds. Chem. Mater..

[72] Reed A.E., Weinstock R.B., Weinhold F. (1985). Natural population analysis. J. Chem. Phys..

[73] Weinhold F., Landis C.R. (2001). Natural bond orbitals and extensions of localized bonding concepts. Chem. Edu. Res. Pr. Euro..

[74] Weinhold F., Landis C.R. (2012). Discovering Chemistry With Natural Bond. Orbitals.

