# Supplementary material for: Diradicalar Character and Ring Stability of Mesoionic Heterocyclic Oxazoles and Thiazoles by Ab Initio Mono and Multi-Reference Methods
Source: Molecules. 2020 Oct 2;25(19):4524. doi: 10.3390/molecules25194524 (PMC7582729; doi:10.3390/molecules25194524)
Supplement: Supplementary file 1 [file molecules-25-04524-s001.pdf]

Diradicalar Character and Ring Stability of Mesoionic Heterocyclic Oxazoles and Thiazoles by Ab Initio Mono and Multi-Reference Methods

By

Antonio João da Silva Filho, Lucinêz da Cruz Dantas, Otávio Luís de Santana *

Chemistry Department, Federal University of Paraiba, João Pessoa 58051-900, Brazil;
* Correspondence: otavio@quimica.ufpb.br; Tel.: +55-(83)-3216-7200

Supplementary Material

Data S01: Optimized geometries at MP2/6-311+G(d) level.

Data S02: HOMO and LUMO orbtials at MP2/6-311+G(d) level optimized geometry.

Data S03: HOMO-LUMO gap (in eV and kJ/mol) for investigated systems from MP2 geometries in gas phase (basis set effect).

Data S04a: Singlet-triplet energy differences (in eV) in gas phase (basis set effect).

Data S04b: Singlet-triplet energy differences (in kJ/mol) in gas phase (basis set effect).

Data S05a: Singlet-triplet energy differences (in eV) in gas phase (triple excitations effect).

Data S05b: Singlet-triplet energy differences (in kJ/mol) in gas phase (triple excitations effect).

Data S06: Orbtials in the CASSCF(12,9) scheme.

Data S07: Energies of states involved in the CASSCF calculation (two with A′ symmetry and two with A″ symmetry) and their respective vertical excitation energies.

Data S08: Occupation numbers of natural bond orbitals, weight of the double excitation configuration and diradical character y_0 (%).

Data S09: Dipole moments (μ) in Debye (D) with 6-311+G(d) and aug-cc-pVDZ basis set in gas-phase.

Data S10: Dipole moments (μ) in Debye (D) with aug-cc-pVTZ basis set.

Data S11: NBO charges on endo and exocyclic atoms groups, with 6-311+G(d) and with aug-cc-pVDZ basis set. The values in parentheses correspond to the endo X and exo Y atoms charges .

Data S12: NBO charges on endo and exocyclic atoms groups, with aug-cc-pVTZ basis set. The values in parentheses correspond to the endo X and exo Y atoms charges .

Data S13: Electrostatic potential maps, calculated at QCISD and CCSD levels, with aug-cc-pVDZ basis set.

Data S01: Optimized geometries at MP2/6-311+G(d) level.

Note: The other geometries were obtained from optimized structures at MP2 level.

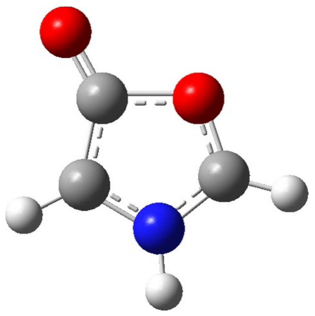

Structure: P1 (RMP2/6-311+G(d) | Charge = 0 | Mult = 1)

Center Number	Atomic Symbol	Coordinates (Angstroms) X	Y	Z	Normal Mode	Frequencies (cm**-1)
1	C	-0.561350	-1.353170	0.000000	1	195.13
2	C	0.000000	0.882940	0.000000	2	380.66
3	C	1.150300	0.073450	0.000000	3	396.35
4	N	0.762220	-1.248150	0.000000	4	504.33
5	H	1.392600	-2.037580	0.000000	5	526.25
6	H	-1.132750	-2.269130	0.000000	6	541.85
7	H	2.182830	0.378050	0.000000	7	660.18
8	O	-1.115660	-0.168230	0.000000	8	665.69
9	O	-0.298330	2.049030	0.000000	9	703.16
					10	923.33
					11	1058.57
					12	1134.25
					13	1171.93
					14	1235.56
					15	1402.94
					16	1491.90
					17	1585.87
					18	1907.75
					19	3305.14
					20	3336.25
					21	3669.74

Structure: P1 (UMP2/6-311+G(d) | Charge = 0 | Mult = 3)

Center Number	Atomic Symbol	Coordinates (Angstroms) X	Y	Z	Normal Mode	Frequencies (cm**-1)
1	C	-1.237609	-0.784148	-0.166488	1	207.18
2	C	0.840962	0.052374	-0.009992	2	229.38
3	C	-0.125581	1.143846	-0.055071	3	476.17
4	N	-1.391393	0.620248	0.146539	4	501.87
5	H	-2.183533	1.089766	-0.273017	5	644.45
6	H	-1.951367	-1.488029	0.249333	6	702.43
7	H	0.109294	2.194679	0.009198	7	733.97
8	O	0.081858	-1.124863	0.043510	8	855.31
9	O	2.030483	0.048540	0.003742	9	861.94
					10	974.89
					11	1031.65
					12	1090.39
					13	1146.43
					14	1197.13
					15	1352.89
					16	1427.50
					17	1455.77
					18	2115.04
					19	3223.03
					20	3320.54
					21	3613.07

Structure: P2 (RMP2/6-311+G(d) | Charge = 0 | Mult = 1)

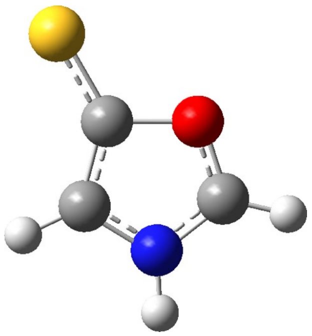

Center Number	Atomic Symbol	Coordinates (Angstroms) X	Y	Z
1	C	-0.305696	-1.828904	0.000000
2	C	0.000000	0.412375	0.000000
3	C	1.240874	-0.246571	0.000000
4	N	1.004047	-1.598194	0.000000
5	H	1.710531	-2.322296	0.000000
6	H	-0.812696	-2.780697	0.000000
7	H	2.233563	0.172479	0.000000
8	O	-0.954180	-0.682154	0.000000
9	S	-0.508585	1.972106	0.000000

Normal Mode	Frequencies (cm**-1)
1	188.60
2	322.35
3	428.62
4	481.71
5	526.23
6	551.46
7	600.42
8	671.25
9	726.92
10	920.22
11	1011.75
12	1095.28
13	1161.04
14	1211.31
15	1289.50
16	1458.12
17	1501.71
18	1583.60
19	3321.29
20	3330.86
21	3652.74

Structure: P2 (UMP2/6-311+G(d) | Charge = 0 | Mult = 3)

Center Number	Atomic Symbol	Coordinates (Angstroms) X	Y	Z
1	C	-1.721423	-0.759291	-0.145850
2	C	0.360088	0.041301	-0.009291
3	C	-0.500883	1.109728	-0.002576
4	N	-1.794951	0.660943	0.100154
5	H	-2.557577	1.189277	-0.300653
6	H	-2.430333	-1.409774	0.359641
7	H	-0.234897	2.156231	-0.002578
8	O	-0.404416	-1.125757	0.026439
9	S	2.012256	0.005831	-0.001418

Normal Mode	Frequencies (cm**-1)
1	199.39
2	268.89
3	318.60
4	534.30
5	557.97
6	610.34
7	816.23
8	837.06
9	906.53
10	982.93
11	1052.15
12	1084.28
13	1141.28
14	1203.69
15	1216.45
16	1333.65
17	1443.51
18	1512.33
19	3197.13
20	3317.17
21	3637.92

Structure: P3 (RMP2/6-311+G(d) | Charge = 0 | Mult = 1)

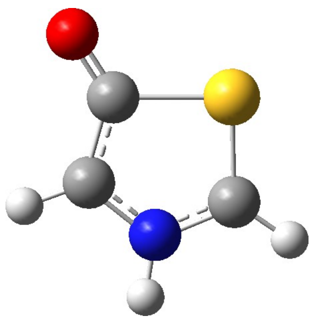

Center Number	Atomic Symbol	Coordinates (Angstroms) X	Y	Z	Normal Mode	Frequencies (cm**-1)
1	C	0.102875	-1.560038	0.000000	1	175.37
2	C	0.000000	1.017062	0.000000	2	311.06
3	C	1.296821	0.430174	0.000000	3	360.72
4	N	1.292764	-0.926696	0.000000	4	476.62
5	H	2.158925	-1.452184	0.000000	5	541.35
6	H	0.024757	-2.637238	0.000000	6	545.11
7	H	2.226130	0.981746	0.000000	7	569.23
8	S	-1.174967	-0.444570	0.000000	8	570.06
9	O	-0.382232	2.173061	0.000000	9	651.79
					10	787.44
					11	907.59
					12	1098.58
					13	1127.08
					14	1206.73
					15	1389.66
					16	1471.49
					17	1551.89
					18	1796.38
					19	3287.08
					20	3291.34
					21	3623.83

Structure: P3 (UMP2/6-311+G(d) | Charge = 0 | Mult = 3)

Center Number	Atomic Symbol	Coordinates (Angstroms) X	Y	Z	Normal Mode	Frequencies (cm**-1)
1	C	-1.494742	-0.303497	-0.246353	1	162.02
2	C	0.953846	0.235516	-0.022561	2	265.40
3	C	0.053811	1.376336	-0.019723	3	399.04
4	N	-1.269061	1.058124	0.174364	4	482.37
5	H	-1.962840	1.735731	-0.113578	5	526.32
6	H	-2.484806	-0.708434	-0.071768	6	583.93
7	H	0.409986	2.396777	0.023578	7	627.41
8	S	-0.082746	-1.279848	0.066749	8	670.89
9	O	2.145943	0.224562	-0.049368	9	728.45
					10	742.28
					11	876.69
					12	1040.03
					13	1064.70
					14	1199.58
					15	1295.11
					16	1451.51
					17	1484.13
					18	2070.78
					19	3243.17
					20	3288.38
					21	3621.32

Structure: P4 (RMP2/6-311+G(d) | Charge = 0 | Mult = 1)

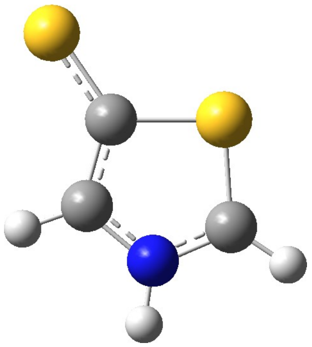

Center Number	Atomic Symbol	X (Angstroms)	Y (Angstroms)	Z (Angstroms)	Normal Mode	Frequencies (cm**-1)
1	C	0.369886	-1.935069	0.000000	1	160.07
2	C	0.000000	0.584820	0.000000	2	264.04
3	C	1.348401	0.152204	0.000000	3	316.14
4	N	1.497835	-1.195676	0.000000	4	397.43
5	H	2.413316	-1.631964	0.000000	5	460.59
6	H	0.372779	-3.015309	0.000000	6	509.97
7	H	2.212779	0.802296	0.000000	7	596.49
8	S	-0.986326	-0.907272	0.000000	8	613.47
9	S	-0.625764	2.119959	0.000000	9	659.45
					10	749.12
					11	887.38
					12	1034.90
					13	1121.86
					14	1192.99
					15	1253.47
					16	1400.25
					17	1503.97
					18	1541.57
					19	3283.23
					20	3289.08
					21	3611.99

Structure: P4 (UMP2/6-311+G(d) | Charge = 0 | Mult = 3)

Center Number	Atomic Symbol	X (Angstroms)	Y (Angstroms)	Z (Angstroms)	Normal Mode	Frequencies (cm**-1)
1	C	1.964789	-0.208348	-0.162390	1	145.88
2	C	-0.511223	0.158043	-0.025717	2	243.88
3	C	0.217272	1.305238	0.008241	3	256.30
4	N	1.553856	1.146354	0.078799	4	418.41
5	H	2.187350	1.910267	-0.107056	5	462.30
6	H	2.921257	-0.504192	0.256577	6	542.97
7	H	-0.217862	2.298549	0.012734	7	621.49
8	S	0.584076	-1.252805	0.028267	8	715.85
9	S	-2.196124	0.049137	-0.005433	9	768.78
					10	878.30
					11	959.16
					12	1026.91
					13	1044.18
					14	1202.46
					15	1256.78
					16	1321.83
					17	1492.22
					18	1628.52
					19	3215.35
					20	3269.15
					21	3658.98

Data S02: HOMO and LUMO orbtials at MP2/6-311+G(d) level optimized geometry.

**HOMO (Symmetry *a*″)**	**LUMO (Symmetry *a*′)**
**P1**	
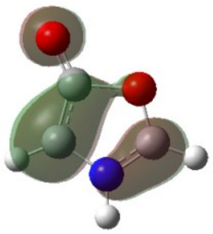	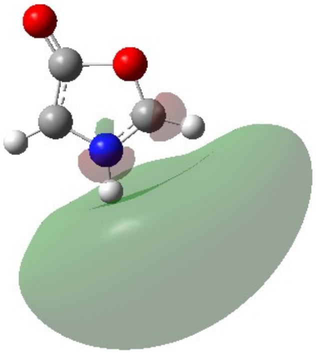
**P2**	
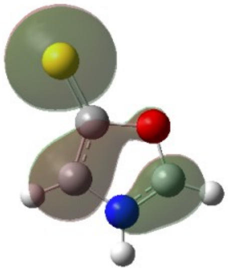	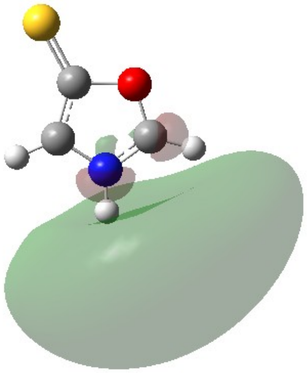
**P3**	
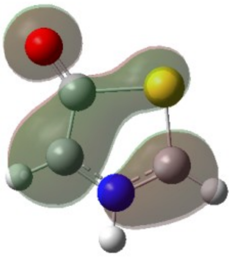	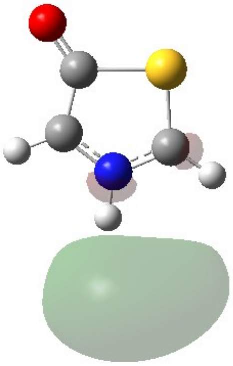
**P4**	
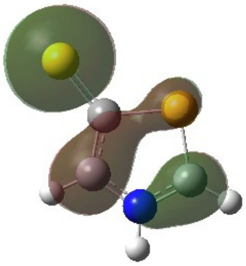	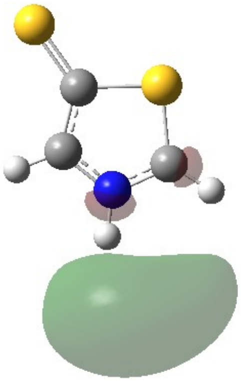

Data S03: HOMO-LUMO gap (in eV and kJ/mol) for investigated systems from MP2 geometries in gas phase (basis set effect).

Method	P1		P2		P3		P4	
	eV	kJ/mol	eV	kJ/mol	eV	kJ/mol	eV	kJ/mol
**BS2**[a]	8.21	792.14	7.35	709.17	8.15	786.36	7.35	709.17
**BS3**[b]	8.09	780.57	7.23	697.59	8.04	775.74	7.23	697.59
**Δ*E***	-0.12	-11.58	-0.12	-11.58	-0.11	-10.61	-0.12	-11.58

[a] BS2: Basis Set aug-cc-pVDZ. [b] BS3: Basis Set aug-cc-pVTZ.

Data S04a: Singlet-triplet energy differences (in eV) in gas phase (basis set effect).

Method	P1		P2		P3		P4	
	B3LYP	MP2	B3LYP	MP2	B3LYP	MP2	B3LYP	MP2
				**Vertical**				
**BS2**[a]	2.89	3.31	2.33	2.99	2.36	3.07	1.89	2.66
**BS3**[b]	2.69	2.92	2.05	2.67	2.06	2.70	1.61	2.30
**Δ*E***	-0.20	-0.39	-0.28	-0.32	-0.30	-0.37	-0.28	-0.36
				**Adiabatic**				
**BS2**[a]	1.89	2.38	1.64	2.30	1.88	2.61	1.55	2.26
**BS3**[b]	2.18	2.76	1.89	2.62	1.99	2.79	1.61	2.35
**Δ*E***	0.29	0.38	0.25	0.32	0.11	0.18	0.06	0.09

[a] BS2: Basis Set aug-cc-pVDZ. [b] BS3: Basis Set aug-cc-pVTZ.

Data S04b: Singlet-triplet energy differences (in kJ/mol) in gas phase (basis set effect).

Method	P1		P2		P3		P4	
	B3LYP	MP2	B3LYP	MP2	B3LYP	MP2	B3LYP	MP2
				**Vertical**				
**BS2**[a]	278.84	319.37	224.81	288.49	227.71	296.21	182.36	256.65
**BS3**[b]	259.55	281.74	197.79	257.62	198.76	260.51	155.34	221.92
**Δ*E***	-19.30	-37.63	-27.02	-30.88	-28.95	-35.70	-27.02	-34.73
				**Adiabatic**				
**BS2**[a]	182.36	229.64	158.24	221.92	181.39	251.83	149.55	218.06
**BS3**[b]	210.34	266.30	182.36	252.79	192.01	269.19	155.34	226.74
**Δ*E***	27.98	36.66	24.12	30.88	10.61	17.37	5.79	8.68

[a] BS2: Basis Set aug-cc-pVDZ. [b] BS3: Basis Set aug-cc-pVTZ.

Data S05a: Singlet-triplet energy differences (in eV) in gas phase (triple excitations effect).

Method	P1		P2		P3		P4	
	**BS1**[a]	**BS2**[b]	**BS1**[a]	**BS2**[b]	**BS1**[a]	**BS2**[b]	**BS1**[a]	**BS2**[b]
				**Vertical**				
**CCSD**	2.77	2.75	2.33	2.36	2.28	2.26	1.81	1.84
**CCSD(T)**[c]	1.87	1.91	1.62	1.70	1.92	1.94	1.55	1.61
**Δ*E***	-0.90	-0.84	-0.71	-0.66	-0.36	-0.32	-0.26	-0.23

[a] BS1: Basis Set 6-311+G(d). [b] BS2: Basis Set aug-cc-pVDZ.
[c] Energies CCSD(T)/6-311+G(d)//CCSD/6-311+G(d) and CCSD(T)/aug-cc-pVDZ//CCSD/aug-cc-pVDZ.

Data S05b: Singlet-triplet energy differences (in kJ/mol) in gas phase (triple excitations effect).

Method	P1		P2		P3		P4	
	**BS1**[a]	**BS2**[b]	**BS1**[a]	**BS2**[b]	**BS1**[a]	**BS2**[b]	**BS1**[a]	**BS2**[b]
				**Vertical**				
**CCSD**	267.26	265.33	224.81	227.71	219.99	218.06	174.64	177.53
**CCSD(T)**[c]	180.43	184.29	156.31	164.03	185.25	187.18	149.55	155.34
**Δ*E***	-86.84	-81.05	-68.50	-63.68	-34.73	-30.88	-25.09	-22.19

[a] BS1: Basis Set 6-311+G(d). [b] BS2: Basis Set aug-cc-pVDZ.
[c] Energies CCSD(T)/6-311+G(d)//CCSD/6-311+G(d) and CCSD(T)/aug-cc-pVDZ//CCSD/aug-cc-pVDZ.

Data S06: Orbtials in the CASSCF(12,9) scheme.

Symmetry *a′*		
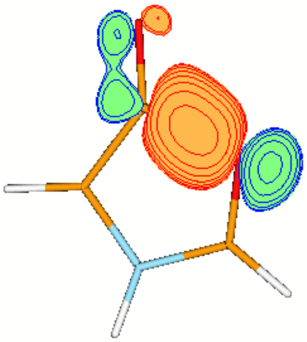 $\sigma_{(C-X)}$	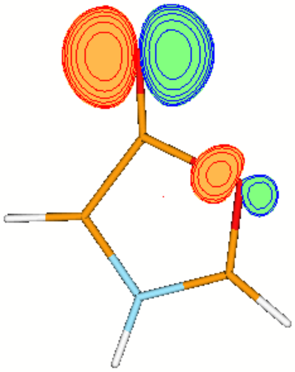 $n_{x(Y)}$	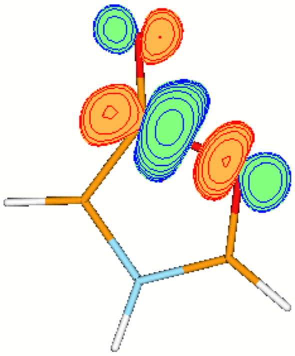 $\sigma^*_{(C-X)}$
**Symmetry *a″***		
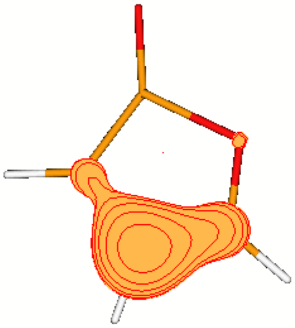 $\pi_{(N-C)}$	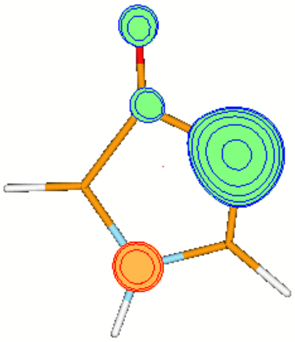 $n_{y(X)}$	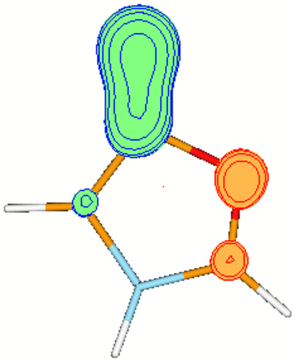 $n_{y(Y)}$
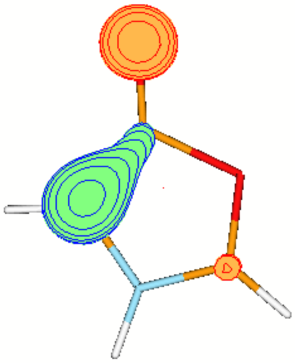 $\pi_{(C-C)}$	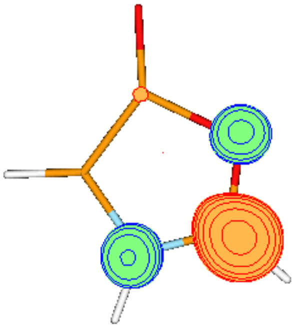 $\pi^*_{(N-C)}$	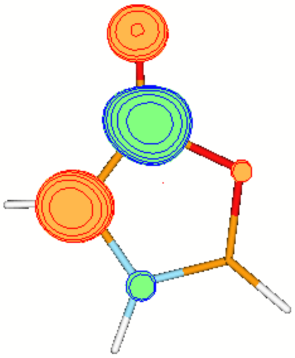 $\pi^*_{(C-C)}$

X and Y: O or S. n_x: Orbital in the plane of the molecule; n_y: Orbital outside the plane of the molecule.

Data S07: Energies of states involved in the CASSCF calculation (two with A′ symmetry and two with A″ symmetry) and their respective vertical excitation energies.

State	E_{abs} [a]	E_{rel} [b]	E_{rel} [c]	State	E_{abs} [a]	E_{rel} [b]	E_{rel} [c]
**CASSCF/6-311+G(d)**							
**Oxazoles**							
	**P1**				**P2**		
1^1 A′	-319.613836497	0.00	0.00	1^1 A′	-642.230873862	0.00	0.00
2^1 A′	-319.465177451	4.05	390.77	2^1 A′	-642.143993839	2.36	227.71
1^1 A″	-319.409769020	5.55	535.49	1^1 A″	-642.149818088	2.21	213.23
2^1 A″	-319.340655160	7.43	716.89	2^1 A″	-642.058053578	4.70	453.48
**Thiazoles**							
	**P3**				**P4**		
1^1 A′	-642.266380297	0.00	0.00	1^1 A′	-964.897906096	0.00	0.00
2^1 A′	-642.130340359	3.70	357.00	2^1 A′	-964.802119288	2.61	251.83
1^1 A″	-642.109583975	4.27	411.99	1^1 A″	-964.807568692	2.46	237.35
2^1 A″	-642.071895444	5.29	510.41	2^1 A″	-964.737219281	4.37	421.64
**CASSCF/aug-cc-pVDZ**							
**Oxazoles**							
	**P1**				**P2**		
1^1 A′	-319.587040702	0.00	0.00	1^1 A′	-642.224069059	0.00	0.00
2^1 A′	-319.436434161	4.10	395.59	2^1 A′	-642.115519593	2.95	284.63
1^1 A″	-319.392190373	5.30	511.37	1^1 A″	-642.109394112	3.12	301.03
2^1 A″	-319.308336619	7.58	731.36	2^1 A″	-642.048767247	4.77	460.23
**Thiazoles**							
	**P3**				**P4**		
1^1 A′	-642.246373156	0.00	0.00	1^1 A′	-964.881971135	0.00	0.00
2^1 A′	-642.108825172	3.74	360.86	2^1 A′	-964.787728093	2.56	247.00
1^1 A″	-642.095319156	4.11	396.55	1^1 A″	-964.792837545	2.43	234.46
2^1 A″	-642.001191121	6.67	643.56	2^1 A″	-964.720214724	4.40	424.54

[a] Absolute Energy (Hartree). [b] Relative Energy (eV). [c] Relative Energy (kJ/mol).
* The lower energy states are highlighted.

Data S08: Occupation numbers of natural bond orbitals, weight of the double excitation configuration and diradical character y_0 (%).

P1 Structure						
			Rigid		Relax	
Phase	Method	Basis	Occupation[a]/ Weight[b]	y_0 (%)	Occupation[a]/ Weight[b]	y_0 (%)
Gas	B3LYP	6-311+G(d)	---[c]	---[c]	---[c]	---[c]
		aug-cc-pVDZ	2.00|0.00	0.00	2.00|0.00	0.00
		aug-cc-pVTZ	2.00|0.00	0.00	2.00|0.00	0.00
	MP2	6-311+G(d)	1.93|0.06	0.23	1.93|0.06	0.23
		aug-cc-pVDZ	1.93|0.06	0.25	1.93|0.06	0.25
		aug-cc-pVTZ	1.93|0.06	0.24	1.92|0.06	0.26
	QCISD	6-311+G(d)	1.91|0.08	0.40	1.91|0.08	0.40
		aug-cc-pVDZ	1.91|0.09	0.42	1.91|0.09	0.42
	CCSD	6-311+G(d)	1.92|0.07	0.32	1.92|0.07	0.32
		aug-cc-pVDZ	1.94|0.07	0.34	1.94|0.07	0.34
	MR-CISD	6-311+G(d)	---[d]	---	0.0117986	2.36
		aug-cc-pVDZ	---[d]	---	0.0076475	1.53
DMSO	B3LYP	6-311+G(d)	---[c]	---[c]	---[c]	---[c]
		aug-cc-pVDZ	---[c]	---[c]	---[c]	---[c]
	MP2	6-311+G(d)	1.93|0.06	0.21	1.93|0.06	0.21
		aug-cc-pVDZ	1.93|0.06	0.23	1.93|0.06	0.23
	QCISD	6-311+G(d)	1.92|0.08	0.35	1.92|0.08	0.35
		aug-cc-pVDZ	1.91|0.08	0.37	1.91|0.08	0.37
	CCSD	6-311+G(d)	1.92|0.07	0.29	1.92|0.07	0.29
		aug-cc-pVDZ	1.92|0.07	0.31	1.92|0.07	0.31

[a] Occupation numbers of frontier natural orbitals (HONO and LUNO). [b] Weight of the double excitation configuration (from HOMO to LUMO, $|c_D|^2$). [c] The structure opens up in the optimization process. [d] In the MR-CISD method, the y_0 is calculated from the ground state of the optimized structure, so that only the relaxed result is calculated.

Data S08: Continuation...

P2 Structure						
**Phase**	**Method**	**Basis**	**Rigid**		**Relax**	
			**Occupation[a]/ Weight[b]**	**y_0 (%)**	**Occupation[a]/ Weight[b]**	**y_0 (%)**
**Gas**	B3LYP	6-311+G(d)	2.00|0.00	0.00	2.00|0.00	0.00
		aug-cc-pVDZ	2.00|0.00	0.00	2.00|0.00	0.00
		aug-cc-pVTZ	2.00|0.00	0.00	2.00|0.00	0.00
	MP2	6-311+G(d)	1.93|0.06	0.24	1.93|0.06	0.24
		aug-cc-pVDZ	1.92|0.06	0.26	1.92|0.06	0.26
		aug-cc-pVTZ	1.92|0.06	0.26	1.92|0.06	0.27
	QCISD	6-311+G(d)	1.91|0.08	0.42	1.91|0.08	0.42
		aug-cc-pVDZ	1.91|0.09	0.44	1.91|0.09	0.44
	CCSD	6-311+G(d)	1.92|0.07	0.33	1.92|0.07	0.33
		aug-cc-pVDZ	1.91|0.07	0.35	1.91|0.07	0.35
	MR-CISD	6-311+G(d)	---[d]	---	0.0080516	1.61
		aug-cc-pVDZ	---[d]	---	0.0081789	1.64
**DMSO**	B3LYP	6-311+G(d)	2.00|0.00	0.00	2.00|0.00	0.00
		aug-cc-pVDZ	2.00|0.00	0.00	2.00|0.00	0.00
	MP2	6-311+G(d)	1.91|0.06	0.21	1.93|0.06	0.21
		aug-cc-pVDZ	1.93|0.06	0.24	1.93|0.06	0.24
	QCISD	6-311+G(d)	1.91|0.07	0.29	1.91|0.07	0.34
		aug-cc-pVDZ	1.91|0.07	0.36	1.91|0.07	0.36
	CCSD	6-311+G(d)	1.92|0.07	0.29	1.92|0.07	0.29
		aug-cc-pVDZ	1.92|0.07	0.31	1.92|0.07	0.31

[a] Occupation numbers of frontier natural orbitals (HONO and LUNO). [b] Weight of the double excitation configuration (from HOMO to LUMO, $|c_D|^2$). [c] The structure opens up in the optimization process. [d] In the MR-CISD method, the y_0 is calculated from the ground state of the optimized structure, so that only the relaxed result is calculated.

Data S08: Continuation...

P3 Structure						
			Rigid		Relax	
**Phase**	**Method**	**Basis**	**Occupation[a]/ Weight[b]**	**y_0 (%)**	**Occupation[a]/ Weight[b]**	**y_0 (%)**
**Gas**	B3LYP	6-311+G(d)	2.00|0.00	0.00	2.00|0.00	0.00
		aug-cc-pVDZ	2.00|0.00	0.00	2.00|0.00	0.00
		aug-cc-pVTZ	2.00|0.00	0.00	2.00|0.00	0.00
	MP2	6-311+G(d)	1.95|0.03	0.09	1.92|0.08	0.35
		aug-cc-pVDZ	1.94|0.03	0.11	1.91|0.08	0.37
		aug-cc-pVTZ	1.94|0.04	0.13	1.91|0.08	0.39
	QCISD	6-311+G(d)	1.94|0.06	0.20	1.94|0.06	0.21
		aug-cc-pVDZ	1.93|0.06	0.22	1.93|0.07	0.23
	CCSD	6-311+G(d)	1.94|0.06	0.18	1.94|0.06	0.18
		aug-cc-pVDZ	1.94|0.06	0.20	1.94|0.06	0.20
	MR-CISD	6-311+G(d)	---[d]	---	0.0093548	1.87
		aug-cc-pVDZ	---[d]	---	0.0083345	1.67
**DMSO**	B3LYP	6-311+G(d)	2.00|0.00	0.00	2.00|0.00	0.00
		aug-cc-pVDZ	2.00|0.00	0.00	2.00|0.00	0.00
	MP2	6-311+G(d)	1.95|0.05	0.12	1.92|0.06	0.27
		aug-cc-pVDZ	1.95|0.04	0.13	1.92|0.07	0.29
	QCISD	6-311+G(d)	1.93|0.07	0.26	1.91|0.08	0.42
		aug-cc-pVDZ	1.93|0.06	0.25	1.91|0.09	0.45
	CCSD	6-311+G(d)	1.94|0.06	0.20	1.92|0.08	0.34
		aug-cc-pVDZ	1.94|0.06	0.21	1.91|0.08	0.36

[a] Occupation numbers of frontier natural orbitals (HONO and LUNO). [b] Weight of the double excitation configuration (from HOMO to LUMO, $|c_D|^2$). [c] The structure opens up in the optimization process. [d] In the MR-CISD method, the y_0 is calculated from the ground state of the optimized structure, so that only the relaxed result is calculated.

Data S08: Continuation...

P4 Structure						
**Phase**	**Method**	**Basis**	**Rigid**		**Relax**	
			**Occupation[a]/ Weight[b]**	**y_0 (%)**	**Occupation[a]/ Weight[b]**	**y_0 (%)**
**Gas**	B3LYP	6-311+G(d)	2.00|0.00	0.00	2.00|0.00	0.00
		aug-cc-pVDZ	2.00|0.00	0.00	2.00|0.00	0.00
		aug-cc-pVTZ	2.00|0.00	0.00	2.00|0.00	0.00
	MP2	6-311+G(d)	1.94|0.03	0.10	1.91|0.07	0.33
		aug-cc-pVDZ	1.94|0.04	0.12	1.91|0.07	0.35
		aug-cc-pVTZ	1.94|0.02	0.09	1.91|0.04	0.22
	QCISD	6-311+G(d)	1.94|0.07	0.22	1.90|0.10	0.54
		aug-cc-pVDZ	1.93|0.07	0.24	1.90|0.10	0.56
	CCSD	6-311+G(d)	1.94|0.06	0.20	1.91|0.08	0.39
		aug-cc-pVDZ	1.93|0.06	0.22	1.91|0.08	0.41
	MR-CISD	6-311+G(d)	---[d]	---	0.0101511	2.03
		aug-cc-pVDZ	---[d]	---	0.0097395	1.95
**DMSO**	B3LYP	6-311+G(d)	2.00|0.00	0.00	2.00|0.00	0.00
		aug-cc-pVDZ	2.00|0.00	0.00	2.00|0.00	0.00
	MP2	6-311+G(d)	1.92|0.06	0.25	1.92|0.06	0.25
		aug-cc-pVDZ	1.92|0.03	0.18	1.92|0.06	0.27
	QCISD	6-311+G(d)	1.91|0.08	0.38	1.91|0.08	0.38
		aug-cc-pVDZ	1.91|0.04	0.23	1.91|0.08	0.41
	CCSD	6-311+G(d)	1.92|0.07	0.33	1.92|0.07	0.33
		aug-cc-pVDZ	1.91|0.04	0.21	1.91|0.07	0.35

[a] Occupation numbers of frontier natural orbitals (HONO and LUNO). [b] Weight of the double excitation configuration (from HOMO to LUMO, $|c_D|^2$). [c] The structure opens up in the optimization process. [d] In the MR-CISD method, the y_0 is calculated from the ground state of the optimized structure, so that only the relaxed result is calculated.

Data S09: Dipole moments (μ) in Debye (D) with 6-311+G(d) and aug-cc-pVDZ basis set in gas-phase.

Structure	Method	Basis	μ_{1r}	μ_{3u}	μ_{3u}
			**Relax**	**Rigid**	**Relax**
**P1**	B3LYP	6-311+G(d)	---[a]	---[a]	---[a]
		aug-cc-pVDZ	6.91	5.22	4.78
	MP2	6-311+G(d)	7.31	6.22	5.28
		aug-cc-pVDZ	6.99	6.03	5.17
	QCISD	6-311+G(d)	7.59	5.42	4.77
		aug-cc-pVDZ	7.27	5.22	4.86
	CCSD	6-311+G(d)	7.71	5.42	4.98
		aug-cc-pVDZ	7.39	5.22	4.85
	MR-CISD	6-311+G(d)	7.48	5.74	---[b]
		aug-cc-pVDZ	7.28	5.48	---[b]
**P2**	B3LYP	6-311+G(d)	8.83	5.00	4.43
		aug-cc-pVDZ	8.66	4.82	4.37
	MP2	6-311+G(d)	8.86	5.48	4.86
		aug-cc-pVDZ	8.69	5.47	4.99
	QCISD	6-311+G(d)	9.39	4.20	3.91
		aug-cc-pVDZ	9.29	4.14	3.93
	CCSD	6-311+G(d)	9.53	4.27	3.91
		aug-cc-pVDZ	9.41	4.14	3.86
	MR-CISD	6-311+G(d)	9.38	5.14	---[b]
		aug-cc-pVDZ	9.23	4.82	---[b]

[a] *The structure opens up in the optimization process.* [b] The MR-CISD geometries in the triplet state have not been optimized.

Data S09: Continuation...

Structure	Method	Basis	μ_{1r}	μ_{3u}	μ_{3u}
			Relax	Rigid	Relax
**P3**	B3LYP	6-311+G(d)	6.51	5.27	4.57
		aug-cc-pVDZ	6.34	5.16	4.54
	MP2	6-311+G(d)	6.51	6.19	5.19
		aug-cc-pVDZ	6.24	6.13	5.13
	QCISD	6-311+G(d)	6.94	5.22	4.46
		aug-cc-pVDZ	6.68	5.13	4.40
	CCSD	6-311+G(d)	7.00	5.23	4.59
		aug-cc-pVDZ	6.73	5.14	4.52
	MR-CISD	6-311+G(d)	6.70	5.45	---[b]
		aug-cc-pVDZ	6.57	5.41	---[b]
**P4**	B3LYP	6-311+G(d)	8.18	5.00	4.42
		aug-cc-pVDZ	8.02	4.91	4.45
	MP2	6-311+G(d)	8.08	5.71	4.95
		aug-cc-pVDZ	7.93	5.78	5.23
	QCISD	6-311+G(d)	8.78	4.41	3.89
		aug-cc-pVDZ	8.71	4.34	3.97
	CCSD	6-311+G(d)	8.92	4.43	3.84
		aug-cc-pVDZ	8.83	4.35	3.96
	MR-CISD	6-311+G(d)	8.39	4.82	---[b]
		aug-cc-pVDZ	8.31	4.60	---[b]

[a] *The structure opens up in the optimization process.* [b] The MR-CISD geometries in the triplet state have not been optimized.

Data S10: Dipole moments (μ) in Debye (D) with aug-cc-pVTZ basis set.

Structure	Method	μ_{1r}	μ_{3u}	μ_{3u}
		Relax	Rigid	Relax
**P1**	B3LYP	6.69	6.77	5.35
	MP2	8.12	6.81	5.41
**P2**	B3LYP	8.58	6.89	4.98
	MP2	10.05	6.04	4.69
**P3**	B3LYP	6.29	7.02	5.04
	MP2	7.44	7.02	5.00
**P4**	B3LYP	7.99	7.20	4.81
	MP2	9.53	6.67	4.63

Data S11: NBO charges on endo and exocyclic atoms groups, with 6-311+G(d) and with aug-cc-pVDZ basis set. The values in parentheses correspond to the endo X and exo Y atoms charges.

Structure	Method	Basis	ENDO ATOMS	EXO ATOMS
**P1**	B3LYP	6-311+G(d)	---[a]	---[a]
		aug-cc-pVDZ	+0.008 (-0.541)	-0.008 (-0.604)
	MP2	6-311+G(d)	+0.029 (-0.601)	-0.028 (-0.728)
		aug-cc-pVDZ	+0.004 (-0.640)	-0.005 (-0.757)
	QCISD	6-311+G(d)	+0.037 (-0.603)	-0.038 (-0.727)
		aug-cc-pVDZ	+0.017 (-0.642)	-0.017 (-0.756)
	CCSD	6-311+G(d)	+0.042 (-0.597)	-0.041 (-0.729)
		aug-cc-pVDZ	+0.019 (-0.637)	-0.019 (-0.759)
**P2**	B3LYP	6-311+G(d)	+0.069 (-0.461)	-0.069 (-0.183)
		aug-cc-pVDZ	+0.061 (-0.480)	-0.061 (-0.188)
	MP2	6-311+G(d)	+0.095 (-0.542)	-0.095 (-0.300)
		aug-cc-pVDZ	+0.077 (-0.573)	-0.077 (-0.300)
	QCISD	6-311+G(d)	+0.116 (-0.538)	-0.116 (-0.320)
		aug-cc-pVDZ	+0.099 (-0.573)	-0.100 (-0.321)
	CCSD	6-311+G(d)	+0.114 (-0.538)	-0.114 (-0.321)
		aug-cc-pVDZ	+0.101 (-0.570)	-0.100 (-0.323)
**P3**	B3LYP	6-311+G(d)	+0.244 (+0.234)	-0.244 (-0.589)
		aug-cc-pVDZ	+0.245 (+0.248)	-0.245 (-0.607)
	MP2	6-311+G(d)	+0.329 (+0.273)	-0.329 (-0.738)
		aug-cc-pVDZ	+0.318 (+0.275)	-0.317 (-0.761)
	QCISD	6-311+G(d)	+0.345 (+0.255)	-0.344 (-0.740)
		aug-cc-pVDZ	+0.336 (+0.256)	-0.335 (-0.764)
	CCSD	6-311+G(d)	+0.343 (+0.257)	-0.344 (-0.739)
		aug-cc-pVDZ	+0.335 (+0.259)	-0.334 (-0.762)
**P4**	B3LYP	6-311+G(d)	+0.436 (+0.428)	-0.436 (-0.166)
		aug-cc-pVDZ	+0.437 (+0.441)	-0.437 (-0.179)
	MP2	6-311+G(d)	+0.514 (+0.459)	-0.514 (-0.294)
		aug-cc-pVDZ	+0.509 (+0.467)	-0.509 (-0.302)
	QCISD	6-311+G(d)	+0.545 (+0.450)	-0.537 (-0.318)
		aug-cc-pVDZ	+0.536 (+0.458)	-0.536 (-0.329)
	CCSD	6-311+G(d)	+0.537 (+0.454)	-0.538 (-0.318)
		aug-cc-pVDZ	+0.538 (+0.463)	-0.537 (-0.329)

[a] The structure opens up in the optimization process.

Data S12: NBO charges on endo and exocyclic atoms groups, with aug-cc-pVTZ basis set. The values in parentheses correspond to the endo X and exo Y atoms charges.

Structure	Method	ENDO ATOMS	EXO ATOMS
**P1**	B3LYP	+0.008 (-0.508)	-0.008 (-0.562)
	MP2	+0.047 (-0.574)	-0.047 (-0.726)
**P2**	B3LYP	+0.092 (-0.428)	-0.092 (-0.189)
	MP2	+0.117 (-0.512)	-0.117 (-0.303)
**P3**	B3LYP	+0.258 (+0.243)	-0.258 (-0.589)
	MP2	+0.335 (+0.276)	-0.335 (-0.739)
**P4**	B3LYP	+0.460 (+0.447)	-0.460 (-0.177)
	MP2	+0.534 (+0.478)	-0.534 (-0.299)

Data S13: Electrostatic potential maps, calculated at QCISD and CCSD levels, with aug-cc-pVDZ basis set.

QCISD

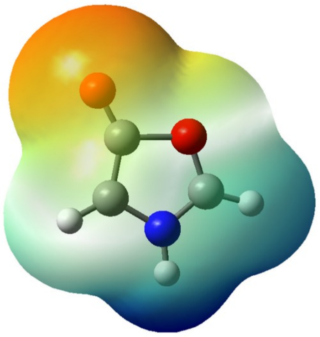

P1

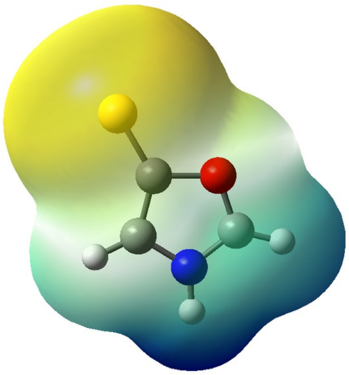

P2

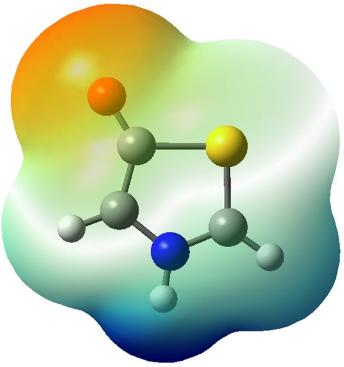

P3

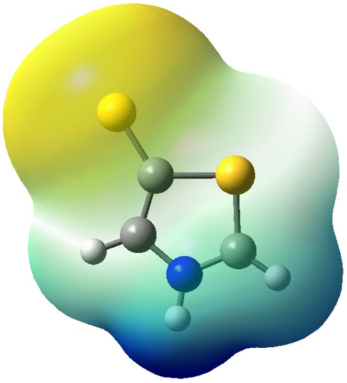

P4

CCSD

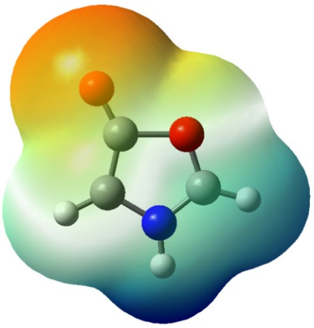

P1

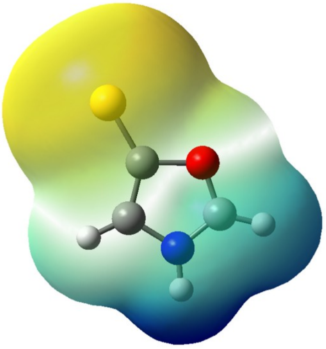

P2

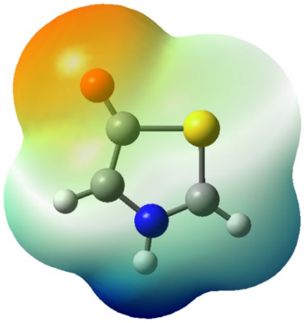

P3

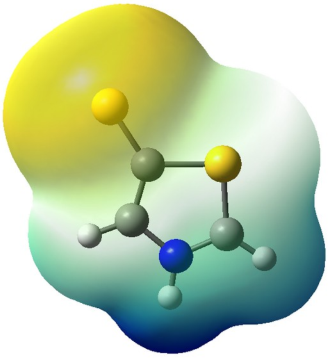

P4
